# Routine Treatment Versus Selective Treatment for Individuals Reporting Contact With Sexual Partners With Chlamydia: A Before-and-After Study

**DOI:** 10.1093/infdis/jiaf107

**Published:** 2025-02-27

**Authors:** Danushi Wijekoon, Marcus Y Chen, Yasmin Hughes, Christopher K Fairley, Catriona S Bradshaw, Jason J Ong, Ivette Aguirre, Eric P F Chow

**Affiliations:** Melbourne Sexual Health Centre, Alfred Health, Melbourne, Victoria, Australia; Melbourne Sexual Health Centre, Alfred Health, Melbourne, Victoria, Australia; School of Translational Medicine, Faculty of Medicine, Nursing and Health Sciences, Monash University, Melbourne, Victoria, Australia; Melbourne Sexual Health Centre, Alfred Health, Melbourne, Victoria, Australia; School of Translational Medicine, Faculty of Medicine, Nursing and Health Sciences, Monash University, Melbourne, Victoria, Australia; Melbourne Sexual Health Centre, Alfred Health, Melbourne, Victoria, Australia; School of Translational Medicine, Faculty of Medicine, Nursing and Health Sciences, Monash University, Melbourne, Victoria, Australia; Melbourne Sexual Health Centre, Alfred Health, Melbourne, Victoria, Australia; School of Translational Medicine, Faculty of Medicine, Nursing and Health Sciences, Monash University, Melbourne, Victoria, Australia; Centre for Epidemiology and Biostatistics, Melbourne School of Population and Global Health, University of Melbourne, Melbourne, Victoria, Australia; Melbourne Sexual Health Centre, Alfred Health, Melbourne, Victoria, Australia; School of Translational Medicine, Faculty of Medicine, Nursing and Health Sciences, Monash University, Melbourne, Victoria, Australia; Melbourne Sexual Health Centre, Alfred Health, Melbourne, Victoria, Australia; Melbourne Sexual Health Centre, Alfred Health, Melbourne, Victoria, Australia; School of Translational Medicine, Faculty of Medicine, Nursing and Health Sciences, Monash University, Melbourne, Victoria, Australia; Centre for Epidemiology and Biostatistics, Melbourne School of Population and Global Health, University of Melbourne, Melbourne, Victoria, Australia

**Keywords:** chlamydia, antibiotic, treatment, contact tracing, resistance

## Abstract

**Background:**

Many international guidelines recommend routine treatment for individuals reporting sexual contact with sexual partners with chlamydia. In October 2019, the Melbourne Sexual Health Centre (MSHC) changed routine treatment of all chlamydia contacts to selective treatment, reserving same-day treatment for those testing positive, unless patients presented with symptoms or with specific reasons.

**Methods:**

We conducted a before-and-after study among chlamydia contacts at MSHC by comparing 12 months before the “routine treatment” period (December 2018 to October 2019) and after the “selective treatment” period (November 2019 to December 2020).

**Results:**

Of the 2843 chlamydia contacts included in the analysis, chlamydia positivity was 31.9% (907/2843). The proportion of contacts who received treatment before test results decreased from 91.1% (1380/1515) to 55.6% (739/1328) (*P* < .0001). We reviewed 232 of the 739 chlamydia contacts in the selective period to determine reasons for treatment; 41.4% (96/232) were treated due to the presence of symptoms. The proportion of those who received treatment and later tested positive did not change between the 2 periods (3% [482/1380] vs 34.2% [253/739]; *P* = .750). The proportion of contacts who received unnecessary treatment (treated but tested negative) did not change between the 2 periods (65.1% [898/1380] vs 65.8% [486/739]; *P* = .750). Of the 60 who did not receive treatment but tested positive subsequently, 7 (11.7%) did not return for treatment, and it did not differ between the 2 periods (*P* = .370).

**Conclusions:**

The selective treatment approach has reduced antibiotic consumption and likely decreased the overall workload of clinic staff by minimizing the need to treat all contacts.

## Teaser

Switching chlamydia contact management from routine to selective treatment could reduce same-day treatment from 91% to 56%. During the selective treatment period, in most contacts, receiving same-day treatment was due to the presence of symptoms.

Sexually transmitted infections (STIs) are increasing in many regions around the world [1, 2], highlighting their continued significance as a global public health challenge. Each year, there are 374 million new cases of STIs, with an estimated 128.5 million cases of *Chlamydia trachomatis* [3], making it the most common bacterial STI globally [4]. International guidelines on managing chlamydia contacts vary. The United States Centers for Disease Control and Prevention recommends evaluation, testing, and presumptive treatment for sexual partners within 60 days of symptom onset or chlamydia diagnosis, and even beyond 60 days for the most recent partner [5]. British guidelines recommend empirical treatment for male cases with urethral symptoms and for contacts within 4 weeks; for all other cases, contacts within the last 6 months should be treated [6, 7]. Although many international guidelines emphasize the importance of testing and confirmatory diagnostics for chlamydia contacts, they continue to recommend presumptive treatment [5–10].

Concerns over antimicrobial resistance in STIs have been increasing [11–13]. Although antibiotic resistance in chlamydia is relatively uncommon, the overprescription and misuse of antibiotics such as doxycycline and azithromycin may foster resistance in other pathogens, such as *Neisseria gonorrhoeae* and *Mycoplasma genitalium*, as well as non-STI bacteria. Recent studies have shown *that N gonorrhoeae* has developed significant resistance to azithromycin, leading to treatment challenges [14–16]. Similarly, *M genitalium* demonstrates increasing resistance to azithromycin, complicating treatment options [17, 18]. The widespread use of these antibiotics in non-STI cases, such as respiratory infections, may further contribute to resistance in non-STI bacteria [19]. Considering that antimicrobial resistance is one of the top global public health threats, driven largely by the overuse and misuse of antibiotics [20], the selective treatment approach aimed to reduce unnecessary antibiotic use. Thus, there is a need to revisit the management of contacts to optimize the use of antibiotics and support antimicrobial stewardship.

The Australian STI guidelines recommend presumptive treatment if sexual contact occurred within the last 2 weeks or if there is a risk that the individual may not be able to receive treatment later due to their circumstances [10]. However, a previous study in Melbourne revealed that only 40% of females, 36% of heterosexual males, and 24% of men who have sex with men (MSM) who reported to be chlamydia contacts tested positive for chlamydia, indicating that more than half of these individuals are not infected and may receive unnecessary antibiotic treatment [21]. To enhance antibiotic stewardship, the Melbourne Sexual Health Centre (MSHC) revised its treatment policy and discontinued routine treatment for all chlamydia contacts on 5 December 2019 and switched to selective treatment management [22]. This study aimed to evaluate the clinical management after switching from routine treatment to selective treatment for individuals reporting contact with sexual partners with chlamydia.

## METHODS

We conducted a before-and-after study using routinely collected clinic records of individuals reporting contact with partners who were diagnosed with chlamydia at MSHC from December 2018 to December 2020. Informed consent was waived because of the retrospective nature of the study. Ethical approval was obtained from the Alfred Hospital Research Ethics Committee (approval number 86/21), ensuring compliance with ethical standards for secondary data use.

MSHC is the largest sexual health service in Australia and the only public sexual health service in Victoria. MSHC provides free human immunodeficiency virus/STI testing and treatment, and no referrals are required. Clients attending MSHC are invited to complete a questionnaire on their demographic and sexual practices via computer-assisted self-interviewing as part of their routine clinical care. These data include demographic information (eg, age, country of birth, and gender) and sexual practices (eg, gender of partners, number of partners, and condom use). A diagnosis code for “chlamydia contact” was used by clinicians to identify clients reporting contact with partners who were diagnosed with chlamydia. The data were stratified into 2 periods: the “routine treatment” period (from 5 December 2018 to 4 December 2019), where all chlamydia contacts were routinely offered treatment before receiving test results; and the “selective treatment” period (from 4 December 2019 to 4 December 2020), where chlamydia contacts were only treated if they tested positive subsequently except when the patients preferred treatment on the same day, presented with urogenital and/or anal symptoms that are related to chlamydia (ie, urethritis with discharge, dysuria, and proctitis among men and cervicitis with vaginal discharge, postcoital bleeding, and proctitis among women), or were unlikely to return for treatment.

For chlamydia contacts, the recommended treatment regimen for uncomplicated genital or oropharyngeal infections was originally a single dose of azithromycin 1 g orally. However, as of 29 October 2020, this was changed to doxycycline 100 mg orally twice daily for 7 days after a randomized controlled trial showing doxycycline is superior to azithromycin for anorectal chlamydia treatment [23]. During the selective treatment period, clients were generally provided with a prescription for chlamydia treatment (hereafter “delayed script”), but they were advised not to fill the prescription or take the medication until they received a positive chlamydia test result. Delayed scripts were only provided during the routine treatment period in special circumstances (eg, clients attending MSHC late and our pharmacy was closed, or upon request).

In this analysis, we included all chlamydia contacts aged 16 years and older, across all genders (male, female, transgender, other), who visited MSHC during the study period. We excluded chlamydia contacts who had already received antibiotic treatment from another service before attending MSHC and those who were also contacts of gonorrhea, syphilis, and/or *Mycoplasma genitalium* as the clinical care and management might vary due to the need to manage STIs other than chlamydia. We also reviewed medical records on a subset of data from both the routine (12 July to 29 November 2019) and selective (13 July to 30 November 2020) treatment periods to examine reasons for treatment given before test results. All contacts were tested for chlamydia on the day of presentation. During the study period, chlamydia was diagnosed using the transcription-mediated amplification Aptima Combo 2 Assay (Hologic Panther system; Hologic, San Diego, California). First-pass urine was collected among all men, and extragenital swabs (ie, anorectal and oropharyngeal) were collected among MSM. Endocervical swabs, vaginal swabs, or first-pass urine were collected among women. The Aptima Combo 2 Assay, consistently used for diagnosing chlamydia in both confirmed cases and their contacts, has a turnaround time of approximately 24–48 hours, with STI results available within 2–5 business days at MSHC [24].

Statistical analyses were conducted in SPSS Statistics version 22 (IBM Corporation, Armonk, New York). Descriptive statistics (eg, median, frequencies, and proportions) were calculated. The 95% confidence intervals (CIs) were calculated using the mid-P exact method with significance set at *P* < .05. Chlamydia positivity was calculated as the number of chlamydia contacts who tested positive for chlamydia divided by the number of chlamydia contacts attending MSHC. The primary outcomes include the proportion of contacts who received treatment before the test results, contacts who received unnecessary treatment (ie, contacts who received treatment but tested negative for chlamydia), and contacts who missed treatment (ie, contacts who tested positive for chlamydia but did not receive treatment due to loss to follow-up) in both periods. The χ^2^ test was used to compare the proportions between the 2 periods.

## RESULTS

From 5 December 2018 to 4 December 2020, 3165 chlamydia contacts attended MSHC. Of these, 322 (10.2%) cases were excluded as they were also contacts of gonorrhea, syphilis, and/or *M genitalium*. The remaining 2843 contacts were included in the study, with 1515 (53.3%) in the routine treatment period and 1328 (46.7%) in the selective treatment period (Table 1). The majority of contacts were MSM (1246/2843 [43.8%]), followed by men who have sex with women only (MSW) (897/2843 [31.6%]), females (693/2843 [24.4%]), and transgender or gender-diverse people (7/2843 [0.2%]). The mean age was 30.0 years (standard deviation, 8.5 years).

**Table 1.** Demographic Characteristics of the Study Population, Stratified by Treatment Period

Characteristics	Routine Treatment Period		Selective Treatment Period		*P* Value
	No.	(%)	No.	(%)	
Total No. of chlamydia contacts	1515		1328		
Chlamydia positive	504	(33.3)	403	(30.3)	.095
Age, y, mean (SD)	29.5	(8.6)	30.2	(8.5)	.036
Country of birth					
Australia	555	(36.6)	505	(38.0)	.170
Overseas	912	(60.2)	766	(57.7)	
Unknown	48	(3.2)	57	(4.3)	
Risk population					
Females	381	(25.1)	312	(23.5)	.432
MSW	487	(32.1)	410	(30.9)	
MSM	644	(42.5)	602	(45.3)	
Transgender and gender-diverse people	3	(0.2)	4	(0.3)	
Ever engaged in sex work	29	(1.9)	37	(2.8)	.302

Data are presented as No. (%) unless otherwise indicated.

Abbreviations: MSM, men who have sex with men; MSW, men who have sex with women only; SD, standard deviation.

Overall, 31.9% (95% CI, 30.2%–33.6%; 907/2843) of chlamydia contacts tested positive for chlamydia, and this did not differ significantly between the routine treatment period (33.3% [95% CI, 30.9%–35.7%]; 504/1515) and the selective treatment period (30.3% [95% CI, 27.9%–32.8%]; 403/1328) (*P* = .095). However, chlamydia positivity varied in different populations (*P* < .0001): Females had the highest positivity (35.8% [95% CI, 32.3%–39.4%]; 248/693), followed by MSW (34.7% [95% CI, 31.6%–37.8%]; 311/897), MSM (27.8% [95% CI, 25.4%–30.4%]; 347/1247), and transgender and gender-diverse people (14.3% [95% CI, .7%–53.0%]; 1/7). However, chlamydia positivity by populations did not differ between the 2 treatment periods (*P* = .432).

The positivity of urogenital chlamydia was 24.8% (95% CI, 23.2%–26.4%; 701/2826), anorectal chlamydia was 21.0% (95% CI, 18.7%–23.4%; 246/1173), and oropharyngeal chlamydia was 3.4% (95% CI, 2.5%–4.4%; 47/1391).

**Figure 1.**
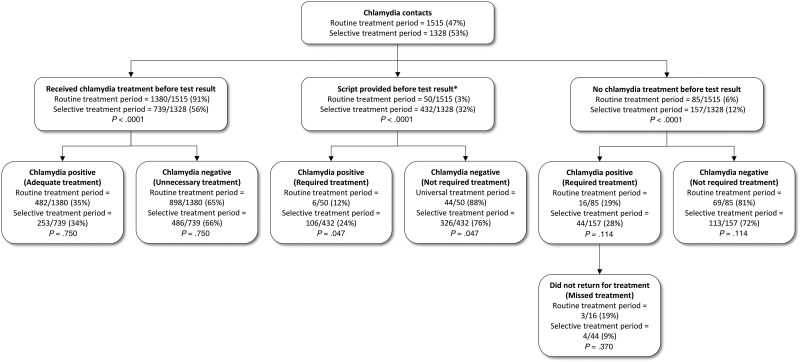
Comparison of chlamydia positivity and treatment among chlamydia contacts between the routine and selective treatment periods. *Script provided to the individual before the test results, and to be dispensed if test is positive for chlamydia.

There was a 39.0% reduction in the proportion of contacts receiving chlamydia treatment before test results, from 91.1% (95% CI, 89.6%–92.4%; 1380/1515) in the routine treatment period to 55.6% (95% CI, 53.0%–58.3%; 739/1328) in the selective treatment period (*P* < .0001; Figure 1).

The proportion of contacts who received treatment before results and who tested positive for chlamydia remained constant in both periods, with 34.9% (95% CI, 32.4%–37.5%; 482/1380) in the routine treatment period and 34.2% (95% CI, 30.9%–37.7%; 253/739) in the selective treatment period (*P* = .750). Similarly, the proportion of contacts who received treatment before results but tested negative for chlamydia also remained consistent in both periods, with 65.1% (95% CI, 62.5%–67.6%; 898/1380) in the routine treatment period and 65.8% (95% CI, 62.3%–69.1%; 486/739) in the selective treatment period (*P* = .750).

The proportion of contacts who received a delayed prescription before test results increased significantly from 3.3% (95% CI, 2.5%–4.3%; 50/1515) in the routine treatment period to 32.5% (95% CI, 30.0%–35.1%; 432/1328) in the selective treatment period (*P* < .0001). Of the 432 contacts who received a delayed prescription in the selective treatment period, only 24.5% (95% CI, 20.6%–28.8%; 106/432) tested positive for chlamydia, suggesting that 75.5% (95% CI, 71.2%–79.4%; 326/432) did not require treatment and these individuals received unnecessary treatment in the routine treatment period.

The proportion of contacts who did not receive any chlamydia treatment before test results increased significantly from 5.6% (95% CI, 4.5%–6.9%; 85/1515) in the routine treatment period to 11.8% (95% CI, 10.2%–13.6%; 157/1328) in the selective treatment period (*P* < .0001). The proportion of contacts who required treatment (ie, contacts who did not receive treatment but tested positive for chlamydia) did not change between the routine treatment period (18.8% [95% CI, 11.6%–28.2%]; 16/85) and the selective treatment period (28.0% [95% CI, 21.4%–35.4%]; 44/157) (*P* = .114). Of the 85 contacts who did not receive treatment in the routine treatment period, most declined to receive treatment on the same day and preferred to wait for test results (56.6%, 48/85), followed by clinician's assessments of the risk of the contacts (16.5%, 14/85); 27.1% (23/85) of cases had unknown reasons. Although these contacts did not receive treatment on the day of attendance, most of these contacts with a positive test result returned to MSHC for treatment (88.3% [95% CI, 78.3%–94.6%]; 53/60). Overall, there were 7 cases (11.7% [95% CI, 5.2%–21.7%]; 7/60) lost to follow-up for treatment, but this proportion did not differ between the 2 periods (*P* = .370).

Of the 739 chlamydia contacts who received treatment before test results in the selective treatment period, we reviewed a subset of 232 cases (31.4%) between July and November 2020 to determine the reasons for treatment (Table 2). The reasons that the 232 contacts were treated before their test were as follows: not recorded (47.0%, 109/232), due to the presence of urogenital and/or anal symptoms (41.4%, 96/232), the patient's preference (eg, upcoming travel, living at a significant distance from the clinic, or work and personal commitments) (8.6%, 20/232), or clinician's preference based on the perception that clients were at higher risk of chlamydia (ie, bisexual individuals, sex workers, or unlikely to return to the clinic for treatment) (3.0%, 7/232). Among the 7 individuals treated based on clinician preference, 28.6% (n = 2) were females, 42.8% (n = 3) were MSW, and 28.6% (n = 2) were MSM. Of the 109 individuals treated for unspecified reasons, 46.8% (n = 51) were MSM, 39.4% (n = 43) were MSW, and 13.8% (n = 15) were females. When treatment was provided to 96 individuals due to the presence of symptoms, 42.7% (n = 41) were MSM, 35.4% (n = 34) were MSW, and 21.9% (n = 21) were females. Of the 20 individuals treated based on patient preference, 55.0% (n = 11) were MSM, 35.0% (n = 7) were MSW, and 10.0% (n = 2) were females.

**Table 2.** Reasons for Chlamydia Treatment and Detection Among Contacts, Stratified by Treatment Period

Reasons	Routine Treatment Period (n = 521)^a^				Selective Treatment Period (n = 232)^b^			
	No. of Contacts		Chlamydia Positivity		No. of Contacts		Chlamydia Positivity	
	No.	(%)	No.	(%)	No.	(%)	No.	(%)
Unknown reasons	328	(63.0)	108	(32.9)	109	(47.0)	25	(22.9)
Presence with symptoms	115	(22.1)	49	(42.6)	96	(41.4)	38	(39.6)
Genital symptoms	109	(94.8)	47	(43.1)	85	(88.5)	35	(41.2)
Rectal symptoms	7	(6.1)	3	(42.9)	13	(13.5)	4	(30.8)
Patient’s preference	15	(2.7)	2	(13.4)	20	(8.6)	7	(35.0)
Clinician's preference	63	(12.1)	24	(38.1)	7	(3.0)	4	(57.1)

^a^Only contacts between July and November 2019 were reviewed.

^b^Only contacts between July and November 2020 were reviewed.

## DISCUSSION

This study evaluated the impact of changing the clinical management for chlamydia contacts, shifting from routine treatment to a selective treatment approach. Our results showed that the selective treatment approach has led to a significant 39% reduction in the number of contacts receiving treatment compared to the routine treatment approach. Given only one-third of contacts tested positive for chlamydia, a selective treatment approach may be more appropriate to optimize antibiotic use to support antimicrobial stewardship.

Our results showed that 56% of chlamydia contacts were treated before the test result in the selective treatment period. We could only identify 1 study evaluating the impact of switching from routine treatment to selective treatment for chlamydia contact. Rasul et al conducted a similar study at the Sydney Sexual Health Centre evaluating the change from empirical antibiotic treatment to nonempirical antibiotic treatment to asymptomatic chlamydia and/or gonorrhea contacts [25]. Rasul et al found that of the asymptomatic chlamydia and gonorrhea contacts, there was a 85% reduction (from 86% to 13%) in the proportion of chlamydia contacts who received empirical treatment. We also saw a 39% (from 91% to 56%) reduction of chlamydia contacts who received treatment before the test results after switching from routine treatment to selective treatment. There are several reasons why the reduction in our study was lower in our study compared with Rasul and colleagues' study. First, their study included only asymptomatic contacts for both chlamydia and gonorrhea, whereas our study focused on chlamydia contacts only and included both asymptomatic and symptomatic individuals. Second, their study was conducted in 2018–2019, but our study spanned 2019–2020 and overlapped with the coronavirus disease 2019 (COVID-19) pandemic, during which time some clients may have been treated before test results to reduce travel and movement.

We found that about only one-third of contacts tested positive for chlamydia, and this is consistent with other studies [21, 24, 26–28]. This further supports that a selective treatment approach for chlamydia contact management should be considered and recommended to reduce unnecessary antibiotic use. A thorough clinician–patient conversation should be encouraged to discuss the potential benefits of delaying treatment until the test results. Other strategies, such as providing a delayed prescription and advising the patients not to take the medication until they receive a positive test result, could also minimize unnecessary treatments. Past studies have demonstrated that delayed antibiotic prescribing effectively reduces overall antibiotic consumption without compromising patient outcomes [29]. There was a significant increase in the proportion of contacts who received a delayed prescription, rising from 3% in the routine treatment period to 32% in the selective treatment period, and >75% of these contacts tested negative, avoiding unnecessary antibiotics. At MSHC, clients who receive delayed prescriptions are explicitly advised by clinicians to take the medication only after being informed their chlamydia test results are positive. Results are usually available within 24–48 hours after sample collection and clients who have a positive test result will be contacted by a follow-up nurse, usually within 8 hours when the test results are available. It is possible that some clients may not follow the advice and take the medication before the test results; however, we are unable to determine this.

In the selective treatment period, 41.4% of contacts received treatment before test results were due to the presence of symptoms. Notably, the chlamydia positivity was higher among those treated at presentation before test results compared to those who received a delayed script for later dispensing (34% vs 24%), which aligns with the clinical rationale that symptomatic individuals may have an elevated likelihood of infection. However, it is important to note that, despite the higher chlamydia positivity, most symptomatic contacts still tested negative for chlamydia. This finding suggests that symptoms alone may not be a reliable indicator of chlamydia positivity and highlights the value of selective treatment protocols in avoiding unnecessary antibiotic use among contacts. Moreover, factors such as patient or clinician preference influenced treatment decisions, albeit to a lesser extent, indicating that non-symptom-driven considerations still played a role in the selective treatment period. These findings support the need for refined criteria in selective treatment practices, balancing timely treatment for true positives to minimize antibiotic use.

Apart from the benefits, we did not see any significant harm after changing this treatment approach to manage chlamydia contacts. There was no significant difference in the proportion of contacts who were lost to follow-up for treatment between the 2 periods (ie, contacts who did not receive treatment before the results but subsequently tested positive for chlamydia and did not return for treatment). However, the role of selective treatment in contacts becomes more complex in cases of coinfection with other STIs, such as gonorrhea, syphilis, and *M genitalium*. While this analysis excluded contacts with multiple STIs, addressing selective treatment in the presence of coinfection and associated complications is critical in clinical practice [30]. Untreated STIs can lead to serious complications, including pelvic inflammatory disease, infertility, and chronic pelvic pain. Managing coinfections often requires broader diagnostic and therapeutic approaches to ensure comprehensive pathogen coverage. Thus, clinical judgment is crucial in these situations to balance effective treatment with minimizing resistance and complications.

There are several limitations in this study. First, this was a before-and-after study using retrospective routinely collected clinical data at a single sexual health clinic. Due to the nature of routinely collected clinical data, some information, especially regarding the reasons for initiating treatment before test results in the selective period, had not been consistently documented. Second, we included the period of the COVID-19 pandemic in our analysis. Victoria had several lockdown restrictions in 2020; however, our service was still open during lockdowns [31]. It is possible that clients who attended MSHC during the COVID-19 pandemic may be different, although we did not see any significant difference in chlamydia positivity between the 2 periods. Some clients in 2020 might request medication to avoid additional travel during the pandemic. Overall, we saw a decrease in the total number of contacts in 2020 during the COVID-19 pandemic compared to 2019, and this may be due to the reduction in casual sexual partners [32]. However, our previous study shows that there was an increase in STI contacts straight after the COVID-19 lockdown, suggesting individuals resumed sexual activity when the lockdown was ceased [33]. Finally, our study did not assess the potential inconveniences for patients associated with the selective treatment approach, particularly the added costs and logistical burdens of follow-up for contacts receiving positive chlamydia test results.

The generalizability of this study's findings to various clinical settings warrants careful consideration. In low- and middle-income countries, where diagnostic microbiology capacity is often limited [34], confirmatory testing for *C trachomatis* may be unavailable. In such settings, empirical treatment is frequently relied upon due to the absence of advanced diagnostic tools. Financial barriers, unregulated availability of antibiotics, and the lack of public health insurance further exacerbate the challenges faced by patients in accessing timely care and follow-up. Poverty and unemployment compound these issues, often resulting in delayed or incomplete treatment for untreated contacts. Addressing these systemic barriers is essential for improving the feasibility and effectiveness of selective treatment approaches in resource-limited environments. Without addressing these systemic obstacles, the selective treatment model proposed in this study may not effectively reduce antibiotic misuse or improve healthcare outcomes in resource-constrained environments [35]. Conversely, in resource-rich settings with advanced diagnostic technologies, selective treatment approaches can be optimized by incorporating rapid molecular diagnostics, such as near-to-patient testing. These point-of-care testing methods enable same-day results, allowing immediate diagnosis and treatment during the same episode of care [36].

Our findings indicate that shifting from routine treatment to selective treatment for *C trachomatis* contacts led to a 39% reduction in the number of contacts receiving treatment, without increasing the proportion of positive cases who missed treatment. This suggests that selective treatment reduces unnecessary antibiotic use without compromising care for positive cases, an essential balance given concerns about antimicrobial resistance. Tailoring selective treatment strategies to specific needs, resources, and constraints of different clinical environments is essential to ensure their broader utility and effectiveness in improving patient outcomes and controlling antimicrobial resistance. Future research should focus on refining selective treatment criteria and exploring additional strategies to optimize antibiotic prescribing practices in sexual health settings.

## Notes

***Acknowledgments.*** We thank Rohit Sasidharan at the Melbourne Sexual Health Centre (MSHC) for his help with extracting electronic data for the study and Finn Mercury at MSHC for his help in assisting with the chart review.

***Author contributions.*** E. P. F. C. and M. Y. C. conceived and designed the study. D. W., under the supervision of E. P. F. C., performed the data analyses and wrote the first draft of the manuscript. E. P. F. C. oversaw the study and provided statistical advice. M. Y. C. and I. A. were involved in revising the internal treatment guidelines at the Melbourne Sexual Health Centre. D. W. and Y. H. performed the chart review. M. Y. C., C. K. F., C. S. B., J. J. O., and E. P. F. C. assisted with data interpretation. All authors revised the manuscript for intellectual content and approved the final version of the manuscript. The corresponding author had full access to all the data in the study and was responsible for the final decision to submit the manuscript for publication.

***Financial support.*** E. P. F. C. and J. J. O. are supported by an Australian National Health and Medical Research Council (NHMRC) Emerging Leadership Investigator Grant (grant numbers GNT1172873 and GNT1193955, respectively). E. P. F. C., M. Y. C., C. K. F., and C. S. B. are each supported by an Australian NHMRC Leadership Investigator Grant (grant numbers GNT2033299, GNT2025840, GNT1172900, and GNT1173361, respectively).
